# The Effects of Diet on the Immune Responses of the Oriental Armyworm *Mythimna separata*

**DOI:** 10.3390/insects14080685

**Published:** 2023-08-03

**Authors:** Lizhen Zhou, Li Ma, Lu Liu, Shaolei Sun, Xiangfeng Jing, Zhiqiang Lu

**Affiliations:** 1 Department of Entomology, College of Plant Protection, Northwest A&F University, Yangling 712100, China; lizhenzhou@nwsuaf.edu.cn (L.Z.); mali890310@gmail.com (L.M.); liulu961108@163.com (L.L.); ssl_0207@163.com (S.S.); jxf_zb@nwafu.edu.cn (X.J.); 2 Key Laboratory of Plant Protection Resources and Pest Management of Ministry of Education, Northwest A&F University, Yangling 712100, China; 3 State Key Laboratory of Crop Stress Biology for Arid Areas, Northwest A&F University, Yangling 712100, China; 4 Key Laboratory of Integrated Pest Management on Loess Plateau of Ministry of Agriculture and Rural Affairs, Northwest A&F University, Yangling 712100, China

**Keywords:** phenoloxidase, antimicrobial peptide, oriental armyworm, nutrition, immune response

## Abstract

**Simple Summary:**

It is known that food nutrients affect hosts immune responses in response to infection. However, the results found in the literature show that the effect of the quantity and quality of foods on immune responses differ across insects. In the current study, we revealed that high protein-to-carbohydrate ratio diets have different impacts on phenoloxidase-mediated melanization and antimicrobial peptide production, but no effects on cellular immune responses in the oriental armyworm, *Mythimna separata* Walker.

**Abstract:**

Nutrients can greatly affect host immune defenses against infection. Possessing a simple immune system, insects have been widely used as models to address the relationships between nutrition and immunity. The effects of high versus low protein-to-carbohydrate ratio (P:C) diets on insect immune responses vary in different studies. To reveal the dietary manipulation of immune responses in the polyphagous agricultural pest oriental armyworm, we examined immune gene expression, phenoloxidase (PO) activity, and phagocytosis to investigate the immune traits of bacteria-challenged oriental armyworms, which were fed different P:C ratio diets. We found the oriental armyworms that were fed a 35:7 (P:C) diet showed higher phenoloxidase (PO) activity and stronger melanization, and those reared on a 28:14 (P:C) diet showed higher antimicrobial activity. However, different P:C diets had no apparent effect on the hemocyte number and phagocytosis. These results overall indicate that high P:C diets differently optimize humoral immune defense responses in oriental armyworms, i.e., PO-mediated melanization and antimicrobial peptide synthesis in response to bacteria challenge.

## 1. Introduction

Immunity and nutrition are two principal factors that cross-modulate host fitness [1]. Immune responses are energetically costly and nutrition plays a key role for energy investment in immune responses [1,2,3,4,5]. The nutritional value of available resources affects the host’s susceptibility and severity to infection [6,7]. For instance, “under” and “over” nutrition can lead to immunodeficiency diseases and increase susceptibility to infection in humans and mice [8,9]. Like vertebrates, insects acquire nutrients from their diet, and the nutritional values of the ingested diets affect the strength of insects’ immune responses [3,6,10,11,12,13,14,15,16].

Insects rely on innate humoral and cellular responses to defend against pathogen invasion [17,18]. Phenoloxidase (PO) and antimicrobial peptides (AMP) are two conserved types of humoral effector molecules in defending against systemic infection in an innate immune system [18,19]. Prophenyloxidase (proPO) is processed to activate PO via a serine protease cascade upon immune challenge. PO then catalyzes the formation of o-quinones, quinones, and other reactive intermediates that kill pathogens and parasitoids [20,21,22,23]. Most AMP expression is strongly induced in fat or epithelial cells via the invasion of pathogens and released into the hemolymph or gut lumen where they exhibit antimicrobial activity [18,19,24,25]. Phagocytosis is one of the conservative and effective cellular immune responses, which is mediated via individual hemocytes to recognize and internalize small foreign microbial intruders, and finally to hydrolytically degrade microbes in the phagolysosome [26,27,28].

Changing the quantity of a single nutritional supplement (e.g., ascorbic acid or selenium) has been implicated in affecting the host’s susceptibility to infection [29,30,31]. Diets that are high or low in nutrition without identifying the key nutritional element also affect the insect’s immune traits. For example, the greater wax moth *Galleria mellonella* larvae and adults that emerged from the larvae reared on a low-nutrition diet exhibited a stronger encapsulation response than those on a high-nutrition diet [11,32]. Studies also revealed that a change in the relative amount of protein and carbohydrate in the diet could affect specific immune responses and survival of the infected hosts [33,34]. Some lepidopteran insects showed that the intake of a high protein-to-carbohydrate (P:C) ratio diet increased their immune responses, including antimicrobial activity, PO activity, and encapsulation response [12,35,36,37]. However, in *Bactrocera* and *Drosophila* flies, the consumption of a low P:C ratio diet resulted in an improved defense against bacteria, showing an improved survival rate and up-regulated key AMP expression [1,38]. These results suggest that the effect of the quantity and quality of diets on immune responses differed across insects. Exploring different insect species will provide us with a broader understanding of insect nutritional immunology.

In this study, we investigated the relationship between dietary nutrition and immunity in the bacteria-challenged oriental armyworm, *Mythimna separata* Walker (Lepidoptera: Noctuidae), which is a widespread polyphagous migratory model insect in China and other Asian countries. We focused on the effects of two key macronutrients, protein (P) and carbohydrate (C), on the immune traits of dead bacteria *Escherichia coli-* and *Micrococcus luteus*-challenged *M. separata*, including PO activity, melanization, antimicrobial activity, AMP production, and phagocytosis. We provided evidence that the immune traits of the oriental armyworm larvae responded in a nutrition-specific manner after bacteria challenging, and took the first attempt to explore the link between immune components and nutritional intake in this species. This lays the foundation for future study on the mechanisms of interaction between nutrition and immunity.

## 2. Materials and Methods

### 2.1. Insects

The oriental armyworm *M. separata* Walker population was provided by Lab of Pest Management, Northwest A&F University, Yang ling, China. *M. separata* is widespread in almost all provinces in China due to its polyphagous feeding habit. In one year, 2–8 generations are produced, varying with latitude [39]. For routine rearing in the laboratory, oriental armyworms were kept in an incubator that was set at 26 ± 1 °C, with 65% ± 5% relative humidity and 14 L: 10 D photoperiod. Newly hatched larvae were reared on fresh corn leaves (about 30 cm plant height) until 6th instar lava started to pupate. Adults were fed with water containing 10% honey under the same environmental conditions. 

### 2.2. Artificial Diet Preparation

Artificial diets were prepared following the previous method [40]. The P:C ratios were made by altering the amounts of protein source (Casein, Albumin, and Peptone) and carbohydrate source (sucrose) in the diet. The other protein and carbohydrate sources were from corn leaf (containing approximately 18.4% protein, 21.52% carbohydrate, 43.2% cellulose, and 0.5% fat on a dry mass basis). These macronutrient concentrations (P + C) made up 42% of the total diets, and other ingredients were the same. We provided full details of the processes and ingredients to prepare the diets in Table A1. 

### 2.3. Insect Growth Measurement

Newly hatched larvae were reared on fresh corn leaves. In order to investigate the effect of a certain diet on the immune responses of oriental armyworms, we referred to previous non-selective experiments (unpublished data) to prepare artificial diets with different ratios of protein (P) and carbohydrate (C): 7:35, 14:28, 21:21, 28:14, and 35:7. On day 1 of the 3rd instar, the larvae were randomly assigned to one of the five artificial diets and housed in perforated Petri dishes (90 mm in diameter). The Petri dishes were cleaned every day, and diets were changed to fresh every other day. On day 2 of the 5th instar, oriental armyworm larvae were weighed individually; there were 20–30 larvae in each group. The larvae were further reared to pupal stage, and the pupae were weighed individually on the third day of the pupal stage. The duration of larval development in the oriental armyworms was also recorded individually. From day 1 of the 3rd instar to day 2 of the 5th instar, oriental armyworm larvae that had similar growth states on different diets were used for the subsequent experiments. 

### 2.4. Dead Bacteria Preparation and Injection

Living pathogens have evolved strategies to resist the host immune response or to compete with the host for nutrients to maintain its own survival [41,42]. Therefore, for simplicity and clarity, we challenged the oriental armyworms with dead bacteria to investigate the immune responses of oriental armyworms reared on artificial diets. Dead bacteria were prepared following a previous method [43]. In brief, *M. luteus* (ATCC 4698; Sigma-Aldrich, St. Louis, MO, USA) and *E. coli* (DH5α) were cultured to logarithmic phase at an optical density (OD600nm) of 0.8~1.0 in LB (Luria-Bertani) medium at 37 °C, with 230 rpm shaking. Bacterial cells were collected via centrifugation at 8000× *g* and washed with sterilized 0.85% NaCl solution three times. Cells were collected again via centrifugation at 8000× *g* in a 50 mL tube and re-suspended in 70% isopropanol. The tube was shaken with 150 rpm at 37 °C on a shaker to fully inactivate the bacteria cells. One hour later, bacterial cells were washed two times with sterilized 0.85% NaCl solution as described above. Centrifugally collected dead *M. luteus* and *E. coli* were re-suspended in sterilized 0.85% NaCl to 6 OD600nm and 4 OD600nm, respectively. The day 2 of the 5th instar oriental armyworm larvae were anesthetized on ice. Each larva was injected with 5 μL bacterial preparations using a micro syringe.

### 2.5. Phenoloxidase Activity Assay

At 6 h, 12 h, and 24 h post injection of bacteria, hemolymph from five larvae per diet were collected into a pre-cooled 1.5 mL Eppendorf tube and centrifuged immediately at 16,000× *g* for 30 s at 4 °C to remove hemocytes (Clark and Strand, 2013). Plasma (2 μL) wasadded to 96-well plate, and 100 μL of 2 mM dopamine was added. Three technical replicates were performed for each sample. After mixing, the absorbance at 490 nm was determined every 30 s for 15 min on a microplate reader (Tecan Pro200, Männedorf, Switzerland). Phenoloxidase activity was shown as the maximum increase in absorbance at 490 nm per minute [44]. The experiment was biologically replicated three times. Data were collected from the mean of technical replicates in each biological replicate.

### 2.6. Melanization Assay of Sepharose Beads in the Hemolymph

For easy observation, QAE Sepharose Fast Flow (QFF) chromatography beads (diameter of 50–150 μm, Sigma) were stained with 0.1% Congo red for 2 h. Dyed QFF beads were washed three times with 50 mM Tris-HCl (pH8.2) and prepared a suspension of 100 μL containing approximately 500 beads. On day 2 of the 5th instar, larvae were injected with a 5 μL suspension of QFF beads. Five oriental armyworms from each diet were dissected at 2 h or 8 h post injection, and beads from each larva were separately transferred into a 96-well plate which contained 50 μL of 50 mM Tris-HCl. Encapsulated and melanized QFF beads were observed under a microscope. In this experiment, beads that were completely covered with melanin to the extent that their own red color could not be observed were called completely melanized beads, while the other beads were called not completely melanized beads. A total of five larvae per diet were analyzed in this assay.

### 2.7. Hemolymph Antimicrobial Activity Assay

At 24 h post injection of bacteria, plasma was collected as described above, heated to 95 °C for 5 min, and then centrifuged at 12,000× *g* for 15 min at 4 °C. Supernatants were then stored at −80 °C. Antimicrobial activity in the supernatants was then measured as previously described with some modifications [45]. *E. coli* was used as the indicator strain and evenly mixed with 10 mL melted LB medium in sterile Petri dishes. Holes (diameter of 1.5 mm) were punched with a 1 mL pipette tip in the LB medium plate. Two μL of the hemolymph supernatant was added to each hole, and each sample was added to three holes. After overnight incubation at 37 °C, the inhibition diameter of the zone was measured. The experiment was biologically replicated three times. Data were collected from the mean of technical replicates in each biological replicate.

### 2.8. Analysis of Lysozyme and Antimicrobial Peptide Gene Expression

Oriental armyworm larvae were injected with bacteria as previously described. Fat bodies from five larvae per diet were collected at 12 h post injection. Total RNA was extracted from the fat body using Tripure reagent (Roche, Basel, Switzerland) following the manufacturer’s protocol. First-strand cDNA was synthesized using Transcriptor First Strand cDNA Synthesis Kit (Roche, Switzerland) according to the manufacturer’s instructions. In the plasma of *E. coli-* and *M. luteus*-infected oriental armyworm, we detected some small molecule weight proteins with antimicrobial activity with LC-MS/MS, including Lysozyme, Cecropin, and Gloverin. Therefore, quantitative real-time PCR (qRT-PCR) was carried out to analyze the expression level of these genes. The prepared cDNA (50 ng/μL) samples were used as templates for qRT-PCR. Amplification reactions were performed on Rotor-Gene Q (Qiagen, Hilden, Germany) using KAPA SYBR FAST qPCR Kit Master Mix (KAPA, Wilmington, MA, USA). The conditions were as follows: 95 °C for 10 min, followed by 40 cycles of 95 °C for 10 s, 58 °C for 20 s, and 72 °C for 20 s. The *M. separata β-actin* gene was used as a reference gene [46]. Three independent experiments were repeated, and the results were calculated using a relative quantitative method (2^−ΔΔCt^) [47]. The experiment was biologically replicated three times. Data were collected from the mean of technical replicates in each biological replicate. Primers used for qRT-PCR and their sequences are listed in Table 1.

### 2.9. Hemocytes Counting

Hemolymph was collected into a pre-cooling tube from a day 2 of the 5th instar larva. In each tube, 1 μL of 1% 1-phenyl-2-thiourea (PTU) was add to prevent the hemolymph from melanizing. Two μL of hemolymph was mixed with 8 μL anticoagulant (4 mM NaCl, 40 mM KCl, 0.1% polyvinylpyrrolidone, 1.9 mM pipes, 4.8 mM citric acid monohydrate, 13.6 mM sodium citrate, 5% sucrose, pH 6.8) and dropped onto a hemocytometer for counting the number under a microscope. In each group, at least eight larvae were individually analyzed.

### 2.10. Phagocytosis Assay

We performed the in vivo phagocytosis assay according to the previous method with some modification [48]. Alexa 594-labeled *E. coli* (K-12 strain) and *Staphylococcus aureus* (Wood strain without protein A) BioParticles conjugate (Invitrogen, Carlsbad, CA, USA) preparations (1 mg/mL) were injected into day 2 of the 5th instar larvae. Two hours later, hemolymph was collected from five larvae per diet and mixed with 1 μL of 1% PTU. One microliter of hemolymph was dropped onto a microslide which contained 100 μL Grace medium (Invitrogen). Slides were kept in the dark for one hour to adhere the hemocytes. The medium was removed and hemocytes were washed with PBS buffer three times, and then fixed with 4% paraformaldehyde for 15 min in the dark and washed three times with PBS buffer. The washed slides were covered with Antifade Mounting Medium (Solarbio, Beijing, China) and scanned using a fluorescent inverted microscope. Phagocytic index = mean fluorescence intensity of hemocytes in fluorescence-positive gate × (number of hemocytes in fluorescence-positive gate/total number of hemocytes). The percent of phagocytic hemocytes = phagocytic hemocytes/total number of hemocytes [49]. At least five views were observed per slide, and the assay was performed four times.

### 2.11. Statistical Analysis

The data in this study were plotted using GraphPad Prism 6.0. Analysis of variance (ANOVA) and Tukey’s multiple comparisons test were used to determine significant differences between groups. 

## 3. Results

### 3.1. Effect of Artificial Diets on the Growth of Oriental Armyworms

**Figure 1:**
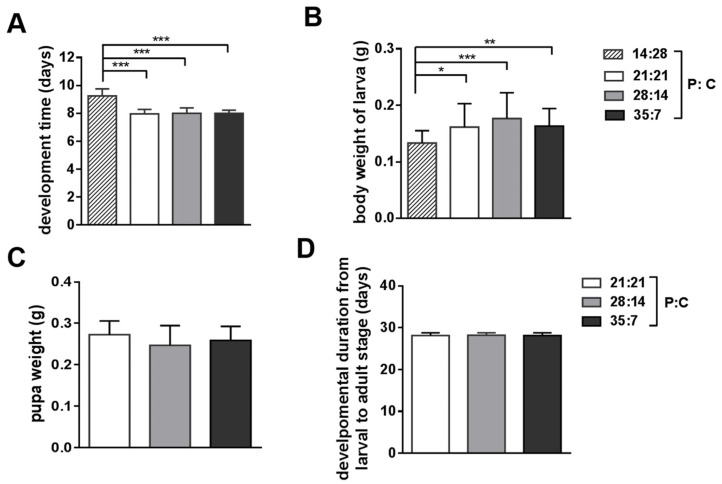
The oriental armyworms reared on different artificial diets showed similar growth. (**A**) The period that larvae developed from day 1 of the 3rd instar to day 2 of the 5th instar was recorded. Body weight (**B**) of oriental armyworm larvae was measured individually on day 2 of the 5th instar. Pupal weight (**C**) was measured at the third day of the pupal stage. The developmental duration from day 1 of the 3rd instar to the first day of the adult stage (**D**) was recorded. Error bars show means ± SEM. One-way analysis of variance (ANOVA) with Tukey’s multiple comparisons test was used to analyze significant differences between two groups (*, *p* < 0.05, **, *p* < 0.01, ***, *p* < 0.001). Analysis without significant difference was not shown on the graph.

**Figure A1:**
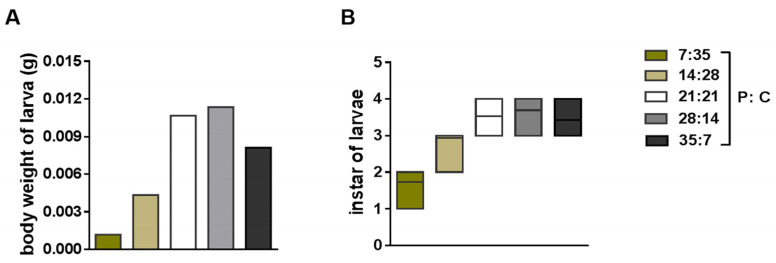
Developmental state of larvae on five kinds of diets. Newly hatched larvae were reared on 7:35, 14:28, 21:21, 28:14, and 35:7 (P:C) diets for seven days, and there were at least 30 larvae in each group. The mean body weight was measured (**A**) and the instar of the larvae was recorded (**B**).

Oriental armyworms that were fed a 14:28 P:C ratio diet took longer to develop from day 1 of the 3rd instar to day 2 of the 5th instar than those on 21:21, 28:14, and 35:7 P:C diets (Figure A1, *F*_3,132_ = 106.8, *p* < 0.0001). Also, as shown in Figure 1B, no significant difference was detected in body weight among oriental armyworms reared on 21:21, 28:14, and 35:7 diets (*F*_3,102_ = 6.926, *p* = 0.0003), while larvae on the 14:28 (P:C) diet were noticeably light in weight. To avoid the effect of nutrient-based trade-offs mentioned above, oriental armyworms fed 21:21, 28:14, and 35:7 P:C artificial diets were selected for subsequent pupal weight and experimental measurement. In addition to this, oriental armyworms fed 21:21, 28:14, and 35:7 (P:C) diets also had similar pupal weight (Figure 1C, *F*_2,28_ = 1.138, *p* = 0.3350) and developmental periods (from day 1 of the 3rd instar to adult stage, Figure 1D, *F*_2,37_ = 0.002989, *p* = 0.9970). We also reared larvae on a diet with a 7:35 (P:C) ratio and found that they grew extremely slowly. We tried to feed newly hatched larvae with these five protein-to-carbohydrate ratio diets, but they did not show similar development states after seven days of feeding (Figure A1). These results indicated that oriental armyworms fed 21:21, 28:14, and 35:7 (P: C) artificial diets from day 1 of the 3rd instar to day 2 of the 5th instar had similar states of growth (body weight and developmental duration). This eliminates nutrient-based trade-offs between growth and immune traits, and ensures that the results in immunity from the following assays are comparable.

### 3.2. Hemolymph PO Activity of Oriental Armyworms

**Figure 2:**
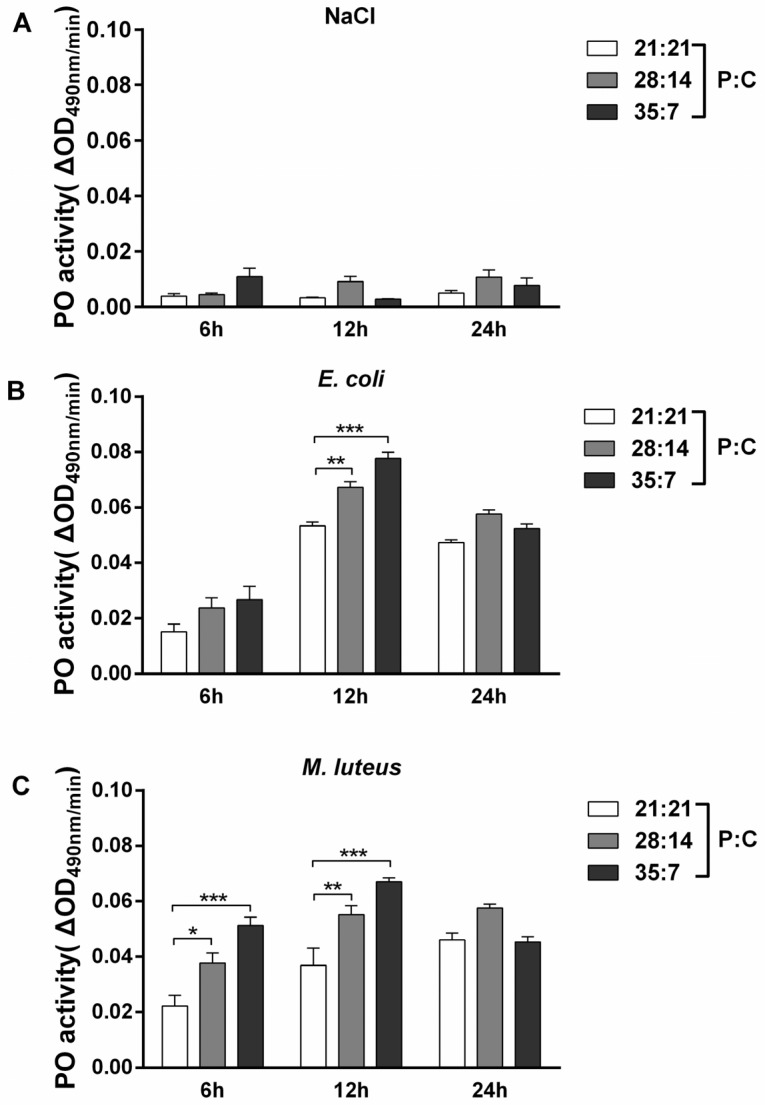
Phenoloxidase activity of the hemolymph from bacteria injected oriental armyworms reared on different diets. The PO (phenoloxidase) activity was assayed in samples from larvae at 6 h, 12 h, and 24 h post injection with 0.85% NaCl (**A**), *E. coli* (**B**), and *M. luteus* (**C**). The control groups were injected with sterilized 0.85% NaCl solution. Error bars represent means ± SEM (*n* = 3). Time points and diets were consider to be two variables, and significant differences between groups were analyzed using two-way ANOVA with Tukey’s multiple comparisons test as post hoc test (*, *p* < 0.05, **, *p* < 0.01, ***, *p* < 0.001).

Conserved PO was measured to explore the effect of diet on the immune responses of *M. separata*. PO activity enhanced from the early injection stage to 12 h post injection, and decreased at the late stage (24 h post injection) (Figure 2). For either bacteria, the lowest PO activity was found in the hemolymph samples from oriental armyworms fed a 21:21 (P:C) diet, and the highest activity in the hemolymph from those fed a 35:7 (P:C) diet at 6 h and 12 h post bacteria injection. The significant difference analysis is shown in Table 2.

### 3.3. Bead Melanization in Oriental Armyworms

**Figure 3:**
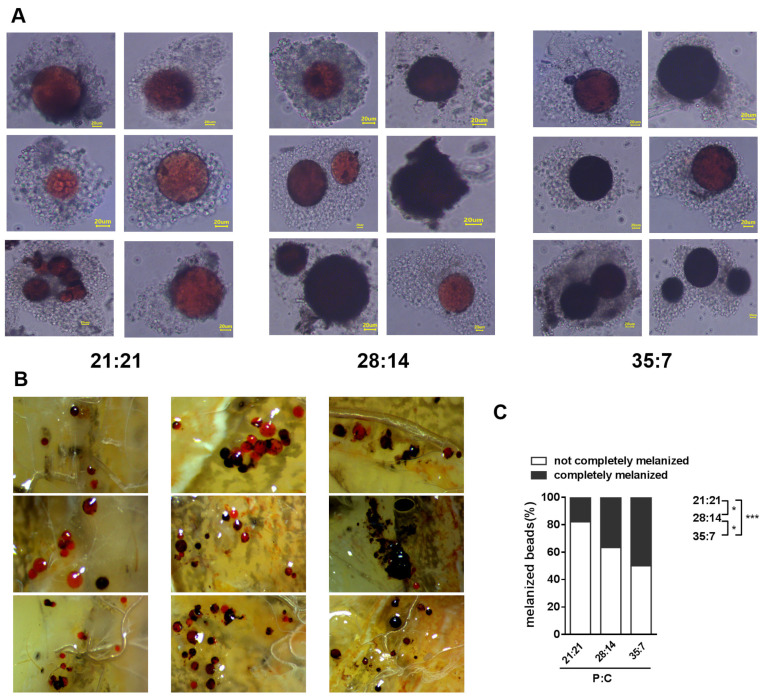
Melanization of sepharose beads in oriental armyworms reared on different diets. Oriental armyworms with sepharose beads inside were dissected at 2 h and 8 h post injection. The bead was viewed under a microscope at 2 h (**A**) and the scale indicated a length of 20 μm. Melanized beads were inspected and counted at 8 h post injection (**B**,**C**). The proportion of beads that were completely melanized was statistically analyzed using one-way ANOVA with Tukey’s multiple comparisons test as post hoc test (*, *p* < 0.05, ***, *p* < 0.001).

PO catalyzes the production of melanin, and melanin physically forms a vesicle sheath on invaders which were encapsulated by specific hemocytes [50]. Thus we analyzed melanin deposition over time by injecting sepharose beads into the oriental armyworm hemocoel. The beads were encapsulated and some were fully melanized at 2 h post injection, particularly beads in oriental armyworms fed 28:14 and 35:7 diets (Figure 3A). More beads were melanized at 8 h post injection in each group, and that about 50% of beads were fully melanized in the oriental armyworms fed a 35:7 (P:C) diet. The modest degree of bead melanization occurred in the oriental armyworms fed with 28:14, and weak melanization was found in the oriental armyworms on 21:21 (P:C) diets (Figure 3B,C, *F*_2,12_ = 15.46, *p* = 0.0005). The degree of melanization is consist with the PO activity of oriental armyworms that were injected with dead bacteria from 6 h to 12 h (Figure 2A,B).

### 3.4. Antimicrobial Activity in the Hemolymph of Oriental Armyworms

**Figure 4:**
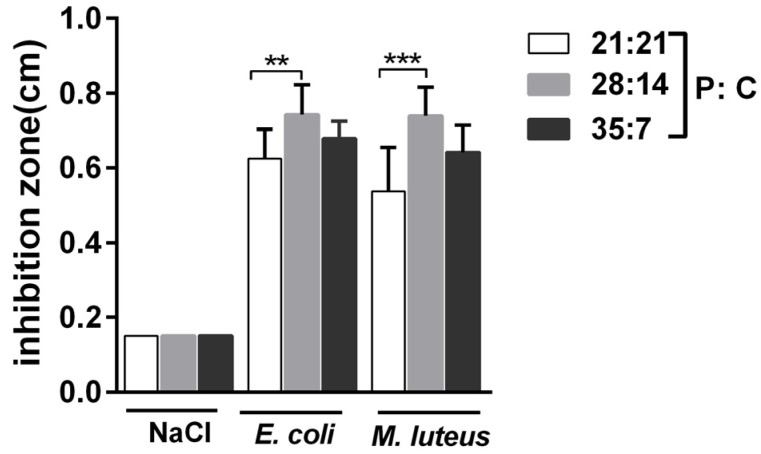
Antimicrobial activity of the hemolymph from bacteria-injected oriental armyworms reared on different diets. Hemolymph was collected from 24 h post bacteria injection and the antimicrobial activity was measured using the inhibitory zone method. The diameter of the holes was 1.5 mm. Error bars represent means ± SEM (*n* = 3). Three independent biological experiments were performed and pairwise comparisons were analyzed with one-way ANOVA with Tukey’s multiple comparisons test (**, *p* < 0.01, ***, *p* < 0.001).

We measured another conserved immune trait, AMPs, to reflect the strength of the immune responses of oriental armyworms (Figure 4). Samples from the oriental armyworms reared on a 28:14 (P:C) diet exhibited the highest activity, those on a 21:21 (P:C) diet exhibited the lowest activity, and larvae on a 35:7 (P:C) diet had moderate activity via either dead *E. coli* or *M. luteus* challenge (for *E. coli* challenge, *F*_2,24_ = 6.317, *p* = 0.0063, for *M. luteus* challenge, *F*_2,24_ = 10.98, *p* = 0.0004).

### 3.5. Lysozyme and AMP Gene Expression in Oriental Armyworms

**Figure 5:**
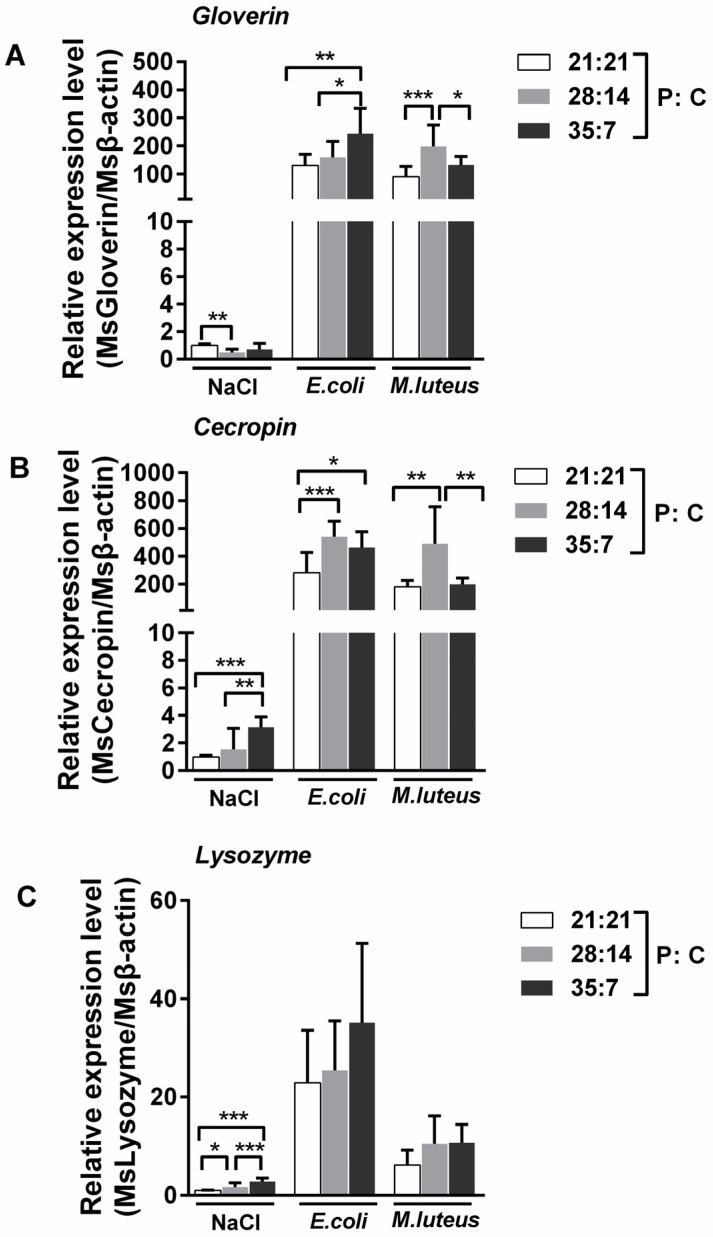
The expression of *Lysozyme* and antimicrobial peptide genes of bacteria-injected oriental armyworms reared on different diets. The fat body was collected from oriental armyworms at 12 h post bacteria injection and mRNA abundance of *Gloverin* (**A**), *Cecropin* (**B**), and *Lysozyme* (**C**) was quantitated using qPCR. The oriental armyworm *β-actin* gene was used as the reference gene. Data were obtained from three independent experiments and fold change was calculated with the 2^−ΔΔCt^ method. NaCl-treated oriental armyworms that were fed a 21:21 P:C ratio diet were chose as the control group, and the others were considered as treatment groups. ΔΔCt = (Ct_target_ − Ct_β-actin_)_treatment group_ − (Ct_target_ − Ct_β-actin_)_control group_. Error bars represent means ± SEM (*n* = 3). Pairwise comparisons were analyzed using one-way ANOVA with Tukey’s multiple comparisons test as post hoc test (*, *p* < 0.05, **, *p* < 0.01, ***, *p* < 0.001).

We further analyzed the expression of the *Gloverin*, *Cecropin,* and *Lysozyme* of oriental armyworms. Saline injection caused the higher expression of *Cecropin* and *Lysozyme* in the oriental armyworms fed a 35:7 (P:C) diet, and the higher expression of *Gloverin* in those on a 21:21 (P:C) diet. Bacterial challenge dramatically increased the expression of these genes, especially *Cecropin*, compared to saline injection (Figure 5). For either bacterial injection, the expression of these genes was lowest in the fat body samples from the oriental armyworms reared on a diet with a 21:21 (P:C) ratio. After *E. coli* injection, the expression of *Gloverin* and *Lysozyme* was higher in the oriental armyworms fed a 35:7 (P:C) diet, and *Cecropin* was higher in those on a 28:14 (P:C) diet. Conversely, after the *M. luteus* injection, the expression of these AMP genes was higher in the oriental armyworms that were fed a 28:14 (P:C) diet. The significant difference analysis is shown in Table 3. The expression level of *Cecropin* is the highest in larvae fed a 28:14 (P:C) diet, which is consistent with the result that these larvae had the highest antimicrobial activity in the hemolymph (Figure 4). 

### 3.6. Hemocyte Number of Oriental Armyworms

**Figure 6:**
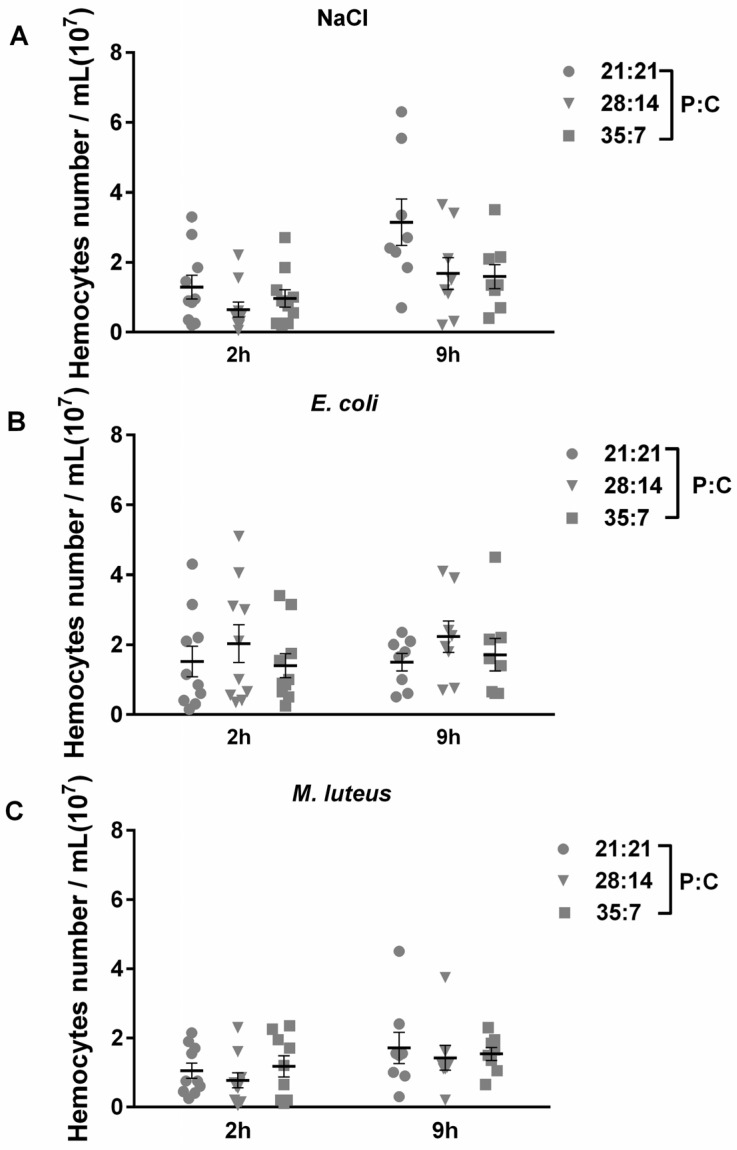
Hemocyte counts of oriental armyworms reared on different diets. On day 2 of the 5th instar, oriental armyworms were injected with dead *E. coli* (**B**) or *M. luteus* (**C**), and oriental armyworms injected with 0.85% NaCl (**A**) were used as control. The number of hemocytes was counted under a microscope at 2 h and 9 h post injection. The results are shown as the total number of hemocytes in one milliliter. Error bars represent means ± SEM (*n* = 10). Time points and diet were consider to be two variables, and two-way ANOVA was used to compare the difference between two groups. Tukey’s multiple comparisons test was used as post hoc test. Analysis without significant difference is not shown on the graph.

Bacterial infection can induce the proliferation of hemocytes, as shown in the black soldier fly (*Hermetia illucens*) at 6 h post infection [51]. Therefore, we investigated whether diet affects hemocyte proliferation in response to bacteria challenge. As shown in Figure 6, P:C ratio diets and time points had no effect on the variation of hemocyte counts in oriental armyworms for either bacterial infection. The significant difference analysis is shown in Table 4. 

### 3.7. Phagocytosis of Oriental Armyworms

**Figure 7:**
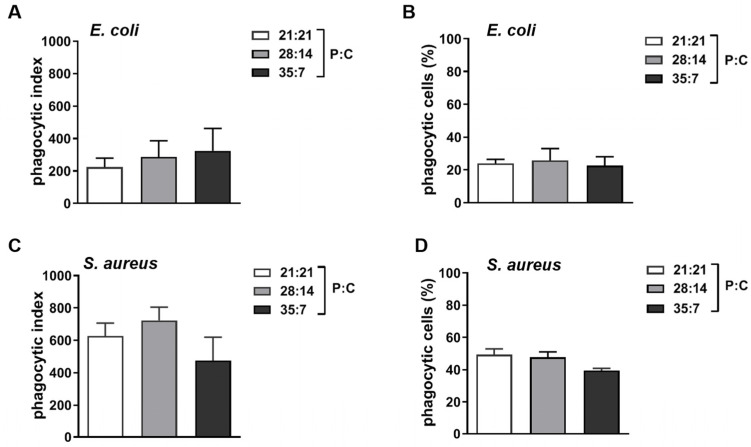
Phagocytosis of fluorescently labeled bacterium in oriental armyworms reared on different diets. Oriental armyworms were injected with fluorescently labeled *E. coli* and *S. aureus*. Hemolymph was collected at 2 h post injection, and fixed hemocytes were analyzed. (**A**,**C**) Phagocytic index = mean fluorescence intensity of hemocytes in fluorescence-positive gate × (number of hemocytes in fluorescence-positive gate/total number of hemocytes). (**B**,**D**) The percent of phagocytic hemocytes = phagocytic hemocytes/total number of hemocytes. Error bars show means ± SEM (*n* = 4). One-way ANOVA was used to compare the difference between two groups and Tukey’s multiple comparisons test was used as post hoc test. Analysis without significant difference is not shown on the graph.

Phagocytosis is stimulated by foreign microbial intruders via individual hemocytes [28]. We explored whether diet affects the phagocytic function of hemocytes in this study. We found the phagocytic index for *S. aureus* was greater than for *E. coli* (Figure 7A,C). The counts for hemocytes that had engulfed fluorescently labeled *S. aureus* (about 50 percent) were more than that for *E. coli* (about 20 percent) at 2 h post injection (Figure 7B,D). However, no significant difference was found between artificial diets regarding either the phagocytic index (for *E. coli*, *F*_2,9_ = 0.2415, *p* = 0.7904, for *S. aureus*, *F*_2,9_ = 1.371, *p* = 0.3021) or the percentage of phagocytic cells (for *E. coli*, *F*_2,9_ = 0.08203, *p* = 0.9219, for *S. aureus*, *F*_2,9_ = 3.187, *p* = 0.0898).

## 4. Discussion

Nutrients have broad impacts on a living organism, and functional immunity is one aspect of the impacts. Proteins and carbohydrates are essential nutrients for organisms, and the P: C ratio can be regulated quantitatively through artificial diet preparation. Insects fed on these diets can be used to explore the impact of quantitative nutritional variation on the physiology of the organism. Previous studies described in the introduction showed that different immune responses boosted by protein-to-carbohydrate ratio diets varied in different insects. In this study, we investigated the specific immune responses of oriental armyworms fed different P:C artificial diets following bacteria challenge. Studies have generally found that there are nutrient-biased trade-offs between immune traits and life history traits, such as growth and longevity [11,32,34]. Individuals that ingested a low-nutrition diet developed more slowly, but they possessed stronger immunity, because they had to invest more energy into coping with more immune challenges which they are likely to encounter during their longer lifespan [11]. Therefore, we considered that the premise of comparing the immunity between individuals is that they have similar growth states. We provided five groups of oriental armyworms as experimental subjects, and we found the larvae fed 14:28 and 7:35 (P:C) artificial diets grew slowly, especially those on the 7:35 (P:C) diet, indicating that low P:C ratio diets, 14:28 and 7:35 diets, provided an insufficient supply of nutrients required for larval growth. Similar results were found in caterpillar *Spodoptera littoralis* [52]. Thus, we selected larvae reared on 21:21, 28:14, and 35:7 (P:C) artificial diets, which showed no obvious difference in growth from the 3rd larval to the adult stage, to compare their immunity.

Although the insect immune system consists of many components, the conserved PO and AMPs have been commonly used to measure the strength of immune responses against different potential pathogens [50,53,54]. We measured the changes in PO activity in the oriental armyworms’ hemolymph at an early stage after bacteria injection, because the PO cascade is a relatively rapid immune response [55]. Our results clearly showed that larvae fed with a high P:C (35:7) diet presented the highest PO activity at 6 h and 12 h post bacteria injection (Figure 2A,B). Similarly, *S. littoralis* and Mormon crickets *Anabrus simplex* showed higher PO activity on high P:C diets [12,56,57]. Melanization is an immune process in which the melanin catalyzed through PO physically encapsulates non-self-invaders [50]. Sepharose beads were strongly melanized in oriental armyworms fed a high P:C (35:7) diet (Figure 3), and this is consistent with the result that a high P:C ratio (35:7) diet was better for oriental armyworms to mount PO activity (Figure 2A,B). Antimicrobial peptides are usually induced in the fat body of insects and released into the hemolymph to defend against systematic infection [19,25]. In our study, the highest antimicrobial activity was detected in the hemolymph from oriental armyworms fed a 28:14 (P:C) diet (Figure 4). In silkworm *Bombyx mori*, the expression level of the *Cecropin* family showed a positive correlation with their antimicrobial activity [58]. Therefore, highly induced Cecropin may contribute the majority of antimicrobial activity in the hemolymph of oriental armyworms fed a 28:14 (P:C) diet, though the larvae fed a 35:7 (P:C) diet showed the highest expression level of *Gloverin* and *Lysozyme* in response to *E. coli* challenge. Similarly, viral-infected *S. littoralis* and bacterium *Bacillus subtilis*-infected Africa armyworm *Spodoptera exempta* on high P:C diets exhibited higher antimicrobial activity [12,35]. 

In our study, oriental armyworms reared on high P:C diets, 35:7 and 28:14, exhibited stronger PO activity and antimicrobial activity. However, a low protein-to-carbohydrate ratio diet improved the survival of *Drosophila* and *B. tryoni* post bacterial infection [1,38]. Therefore, the dietary nutrients required (protein-to-carbohydrate ratios) to maximize an immune response vary among different insects. Infected insects can regulate the allocation of internal nutrients in a given diet so that they present different strengths of immune responses [34]. Although oriental armyworms have similar growth on these three P:C ratio diets, their bodies are likely to differ in internal nutrients, which might be the reason why they mounted immune responses with varying strength. In the present study, we restricted oriental armyworms to a certain P:C ratio diet without diet choice. It is most likely that bacterial challenged oriental armyworms allocated internal nutrients to optimize PO activity and antimicrobial activity. We found that oriental armyworms fed a 35:7 (P:C) diet showed higher PO activity and stronger melanization. The synthesis of nitrogen-rich melanin may require substantial nitrogen and protein, because a sufficient protein resource is available to provide tyrosine (substrate) and PO (catalytic enzyme), which are vital components for melanin production [34,59]. The high P:C diet may supply the optimal ratio to optimize PO activity and melanization in oriental armyworms. Whilst the antimicrobial activity requires protein supplement [34], oriental armyworms fed a 28:14 (P:C) diet showed higher antimicrobial activity rather than those fed a 35:7 (P:C) diet. Previous studies have found evidence for the trade-off between antimicrobial activity and PO activity in insect immune systems [34,60]. It is possible that oriental armyworms fed a 35:7 (P:C) diet invested much more energy in constructing PO activity, resulting in slightly lower antimicrobial activity under limited resources. Moreover, a 28:14 (P:C) diet may be the most appropriate ratio for oriental armyworms to maximize antimicrobial activity against dead bacterial challenge. Moreover, we found dietary P:C ratios had no effect on hemocyte count and phagocytosis. We speculate that the reasons are the following: the majority of hemocytes are phagocytic in insects and phagocytosis is a renewable process [28,61]. In addition, we used dead bacteria to trigger phagocytosis and this eliminated bacterial virulence to hemocytes. Phagocytosis relies on the endocytosis of hemocytes and does not consume as much protein to synthesize humoral immune effectors. Thus, we found no effect of nutritional diets on hemocyte count and activity. Unlike the lepidopterans and flies mentioned in the introduction, we found no certain P:C ratio diet could optimize these immune traits in *M. separata*. How specific immune responses are specifically driven by the amount of protein and carbohydrate intakes requires further investigation. 

Antibiotic abuse has generated many drug-resistant microbes. Antimicrobial peptides can destroy many types of pathogens and the risk of resistance is low [62]. Antimicrobial peptides derived from insects can be used as antibacterial biological ingredients [63]. In this experiment, we found that antimicrobial activity of oriental armyworms fed a 28:14 P:C ratio diet was strong, which may provide a basis for the exploration and utilization of insect antimicrobial peptide resources. Furthermore, insects are an important source for human food and animal feed [64]. We found oriental armyworms fed 21:21, 28:14, and 35:7 P:C ratio diets grew well; this may facilitate the development of armyworms as a feed resource.

## 5. Conclusions

It is known that nutrients affect host immune responses in response to infection. Our results suggest that diets with a high ratio of protein to carbohydrate (28:14 and 35:7 diets) have different impacts on phenoloxidase-mediated melanization and antimicrobial peptide production, but no effects on cellular immune responses in the oriental armyworm. Our study provides evidence supporting the fact that the immune traits of oriental armyworm larvae under similar growth conditions responded in a nutrition-specific manner to bacteria challenge. Accordingly, how a certain P:C ratio diet maximizes an insect’s immune response remains unclear; it needs to be investigated in the future. This is the first attempt to explore the link between immunity and nutrition in the oriental armyworm, and lays a foundation for further study on the mechanism.

## Figures and Tables

**Table 1:** Primers for antimicrobial peptides of *M. separata*.

Gene	Primer Sequence (5′→3′)
*Msβ-actin*	F: TTCAACACACCCGCCATGTA R: CGTAACCCTCGTAGATGGGC
*MsCecropin*	F: GTGCGTTTGCTTCGTCATCA R: GGCTTTGGTGAGCACGTCTA
*MsLysozyme*	F: GTTCTCCACCAGGCATACCC R: TGCGACGATTTGCTTGGTTG
*MsGloverin*	F: TGGGGCTTGTCATGTCCAAA R: TGACTCTCTCAGGTGATGGAGT

**Table 2:** Two variables statistics for time and diet treatment effects on the PO activity of oriental armyworms. *p* ≤ 0.05 indicates statistical significance.

Source of Variation	d.f.	SS	MS	*F*	*p*
NaCl injection					
Time × Diet	4	0.0002872	7.181 × 10^−5^	3.497	0.0134
Time	2	8.056 × 10^−5^	4.028 × 10^−5^	1.962	0.1511
Diet	2	0.0001753	8.765 × 10^−5^	4.268	0.0193
Residual	51	0.001047	2.053 × 10^−5^		
*E. coli* challenge					
Time × Diet	4	0.0007592	0.0001898	4.702	0.0026
Time	2	0.01956	0.009780	242.3	<0.0001
Diet	2	0.002073	0.001036	25.67	<0.0001
Residual	51	0.002059	4.037 × 10^−5^		
*M. luteus* challenge					
Time × Diet	4	0.002452	0.0006131	7.833	<0.0001
Time	2	0.002953	0.001477	18.87	<0.0001
Diet	2	0.004398	0.002199	28.10	<0.0001
Residual	54	0.004227	7.827 × 10^−5^		

**Table 3:** Univariate statistics for diet treatment effects on the expression of Lysozyme and AMP genes in oriental armyworms. *p* ≤ 0.05 indicates statistical significance.

	Treatment	NaCl Injection	*E. coli* Challenge	*M. luteus* Challenge
Gene				
*Gloverin*		*F*_2,24_ = 6.775	*F*_2,24_ = 7.249	*F*_2,24_ = 10.00
		*p* = 0.0046	*p* = 0.0034	*p* = 0.0007
*Cecropin*		*F*_2,24_ = 11.71	*F*_2,24_ = 10.23	*F*_2,24_ = 10.80
		*p* = 0.0003	*p* = 0.0006	*p* = 0.0005
*Lysozyme*		*F*_2,33_ = 25.64	*F*_2,24_ = 2.360	*F*_2,24_ = 3.290
		*p* < 0.0001	*p* = 0.1159	*p* = 0.0546

**Table 4:** Two variables statistics for time and diet treatment effects on the hemocyte count of oriental armyworms. *p* ≤ 0.05 indicates statistical significance.

Source of Variation	d.f.	SS	MS	*F*	*P*
NaCl injection					
Time × Diet	2	3.459	1.730	1.308	0.2798
Time	1	18.34	18.34	13.87	0.0005
Diet	2	11.87	5.934	4.488	0.0163
Residual	48	63.47	1.322		
*E. coli* challenge					
Time × Diet	2	0.2546	0.1273	0.07581	0.9271
Time	1	0.3612	0.3612	0.2151	0.6449
Diet	2	4.250	2.125	1.265	0.2914
Residual	48	80.61	1.679		
*M. luteus* challenge					
Time × Diet	2	0.2566	0.1283	0.1665	0.8471
Time	1	4.100	4.100	5.320	0.0255
Diet	2	0.8762	0.4381	0.5685	0.5702
Residual	47	36.22	0.7706		

## Data Availability

The data presented in this study are available from the corresponding author upon reasonable request.

## Appendix B

**Table A1:** The processes and ingredients to prepare P:C ratio diets in this study. Amounts of ingredients in the 200 g diet are listed below.

Ingredients	7:35	14:28	21:21	28:14	35:7
Step 1					
Casein (g)	0.3831	1.876	3.369	4.862	6.355
Albumin (g)	0.1277	0.625	1.123	1.621	2.118
Peptone (g)	0.1277	0.625	1.123	1.621	2.118
WDCYH salt (g)	2	2	2	2	2
Cellulose (g)	9.71	9.71	9.71	9.71	9.71
Cysteine HCl (g)	0.2	0.2	0.2	0.2	0.2
Choline chloride (g)	0.2	0.2	0.2	0.2	0.2
Myo-inositol (g)	0.08	0.08	0.08	0.08	0.08
vitamin mix (g)	0.325	0.325	0.325	0.325	0.325
Cholesterol (g) (dissolved in 14 mL Chloroform)	0.03737	0.03737	0.03737	0.03737	0.03737
Ingredients are placed in a fume hood until the chloroform completely evaporated.					
Step 2					
Methyl4-hydroxybenzoate (g)	0.39	0.39	0.39	0.39	0.39
L-ascorbic acid (g)	0.8	0.8	0.8	0.8	0.8
Chlortetracycline HCl (g)	0.00694	0.00694	0.00694	0.00694	0.00694
Streptomycin (g)	0.00694	0.00694	0.00694	0.00694	0.00694
Sorbic acid	0.2	0.2	0.2	0.2	0.2
Sucrose (g) (dissolved in 50 mL water)	10.652	8.164	5.675	3.187	0.698
Corn leaf powder (g)	10	10	10	10	10
Mix the ingredients from step 1 and step 2.					
Step 3					
alcohol phase solution					
dl-alpha-tocopherol acetate (mL)	0.02	0.02	0.02	0.02	0.02
alpha-linolenic acid (mL)	0.054	0.054	0.054	0.054	0.054
Linoleie acid (mL)	0.11	0.11	0.11	0.11	0.11
Menadione (g)	0.001	0.001	0.001	0.001	0.001
Cholecalciferol (g)	0.001	0.001	0.001	0.001	0.001
100% ethanol (mL)	2	2	2	2	2
aqueous phase solution					
Biotin (g)	0.00004	0.00004	0.00004	0.00004	0.00004
D-Pantothenic acid (g)	0.002	0.002	0.002	0.002	0.002
Cobalt chloride (g)	0.0005	0.0005	0.0005	0.0005	0.0005
Folic acid (g)	0.0005	0.0005	0.0005	0.0005	0.0005
Nicotinic acid amide (g)	0.002	0.002	0.002	0.002	0.002
Pyridoxine HCl (g)	0.0005	0.0005	0.0005	0.0005	0.0005
Riboflavin (g)	0.001	0.001	0.001	0.001	0.001
Sodium molybdate (g)	0.0005	0.0005	0.0005	0.0005	0.0005
Thiamine HCl (g)	0.0005	0.0005	0.0005	0.0005	0.0005
Vitamin B12 (g)	0.000004	0.000004	0.000004	0.000004	0.000004
Zinc acetat (g)	0.001	0.001	0.001	0.001	0.001
H2O (mL)	12	12	12	12	12
Add 2 mL alcohol phase solution and 12 mL aqueous phase solution into mixture above.					
Step 4					
Agar (g)	3	3	3	3	3
H2O (mL)	150	150	150	150	150
Boiled agar solution were mixed with diet ingredients above.					
37% formaldehyde (mL)	0.416	0.416	0.416	0.416	0.416
